# Spectral and Acoustic Characterization of Nanoenergetic Devices Based on Sodium Perchlorate-Impregnated Porous Silicon

**DOI:** 10.3390/nano15211672

**Published:** 2025-11-03

**Authors:** Abel Apaza Quispe, Ana C. Bueno Borges, Walter Jaimes Salcedo

**Affiliations:** Escola Politécnica, Universidade de São Paulo, São Paulo 5508-220, Brazil; anacb.bueno@usp.br

**Keywords:** nanoporous silicon, sodium perchlorate, nano-explosive, optical spectroscopy, nanomedicine

## Abstract

This work reports the controlled synthesis and characterization of nanoenergetic composites composed of porous silicon (PS) impregnated with sodium perchlorate (NaClO_4_) for precision energy-release applications. PS films were fabricated by electrochemical anodization of p-type silicon (10–20 Ω·cm), with systematic variation in current density (50–200 mA cm^−2^) and anodization time (10–25 min) to tailor pore morphology. The energetic behavior of the composites was evaluated through thermal ignition tests, optical emission spectroscopy (300–1000 nm), acoustic analysis (0–500 Hz), and high-speed imaging. Optimal energy release was obtained for PS films anodized at 100 mA cm^−2^ for 15–20 min, attributed to their hierarchical pore architecture that facilitated complete oxidant infiltration. Overall, this work provides additional insights beyond previous reports by correlating the explosive efficiency with both anodization time—linked to PS film thickness—and current density—associated with porosity. A portable multispectral optical system with fiber-optic access to the explosion chamber was developed for in situ characterization, offering a safe and versatile approach for measurements in explosive environments. To the best of our knowledge, no prior studies have analyzed the correlation between the acoustic signatures and explosion intensity in PS–NaClO_4_ systems as proposed here.

## 1. Introduction

Porous silicon (PS) has emerged as a transformative platform in the field of nanoenergetic materials, particularly for applications demanding precise and controllable energy release. The first evidence of explosive behavior in PS was reported in 1992, when mixtures of PS with oxidizers such as potassium nitrate or nitric acid exhibited strongly exothermic reactions [1]. Subsequent investigations confirmed that these reactive properties are preserved under cryogenic conditions [2,3,4] and at room temperature when appropriate oxidizers are employed [5] highlighting the robustness of PS-based energetic systems.

The exceptional behavior of PS arises from its unique nanostructure—an interconnected porous network with porosities up to 90% and nanocrystals smaller than 10 nm—which provides a highly reactive environment for controlled combustion [6,7,8], Extensive research has demonstrated that the energetic efficiency of PS composites is determined by three interdependent factors: (i) the morphological characteristics of the PS film, especially the specific surface area (exceeding 250 m^2^ g^−1^) and pore-size distribution [2,7,9]; (ii) the physicochemical nature of the oxidizer and its infiltration capacity within the porous matrix [10]; and (iii) the ignition protocol, which governs the combustion kinetics [11,12]. Understanding and optimizing these factors require a precise materials-engineering approach.

Over the last decade, efforts to identify efficient oxidants have highlighted sodium perchlorate (NaClO_4_) as one of the most promising candidates. NaClO_4_ exhibits rapid oxygen-release kinetics (below 10 ms) and a high energy density (≈3.5 kJ g^−1^) [13,14]. When combined with PS, it forms highly reactive composites capable of reaching combustion temperatures above 1600 °C [15,16]. Comparative analyses show that NaClO_4_ outperforms other perchlorates—such as Ca(ClO_4_)_2_ and KClO_4_—in terms of reaction rate and energy released per unit mass [17,18,19], establishing it as a benchmark oxidizer for micropropulsion and precision detonation applications [20].

The present study aims to advance the understanding of PS–NaClO_4_ nanoenergetic systems by addressing three major objectives: (1) optimizing fabrication parameters through systematic control of current density (50–200 mA cm^−2^) and anodization time (10–25 min) to achieve hierarchical pore architectures [21,22,23]; (2) employing multimodal diagnostic techniques—including optical emission spectroscopy (300–1000 nm), high-speed thermographic imaging (≥100,000 fps), and acoustic signal analysis (0–500 Hz)—to characterize combustion dynamics [24,25,26]; and (3) quantitatively validating NaClO_4_ as an optimal oxidizer through direct comparison with other perchlorates [17,19,27].

Calorimetric and pressure analyses indicate that NaClO_4_ generates higher oxygen output and less solid residue than KClO_4_ or Ca(ClO_4_)_2_, resulting in cleaner reactions and approximately 30% higher peak pressures in nSi/NaClO_4_ mixtures [28,29]. These properties make PS-NaClO_4_ composites particularly attractive for self-destructing nanoimplants, microcauterization systems, and micropropulsion technologies. Furthermore, their integration with electrochemical or electro-osmotic MEMS micropumps enables in situ delivery of nanoenergetic doses at microliter precision, minimizing systemic effects [30]. Hybrid configurations—such as image-guided (US/MRI) nanopumping coupled with implantable micropumps—demonstrate additional potential for combined therapies (e.g., tumor ablation with localized drug release) due to NaClO_4_’s high reactivity, low toxicity, and compatibility with polymeric matrices [31].

In this work, PS nanoenergetic films were fabricated under controlled anodization conditions, impregnated with oxidizers, and ignited in confined environments [22,23,32], Optical, thermal, and acoustic emissions were analyzed in the time–frequency domain to correlate structural parameters with energetic performance, following the methodology proposed by Müller [33] to optimize oxidant combustion and energy release [12,34,35].

Samples anodized at 100 mA cm^−2^ for 15–20 min exhibited the highest performance, achieving complete energy release within 140–335 ms. Emission spectra revealed characteristic bands between 400 and 750 nm (O-I, Na-I transitions) and 800–950 nm (thermal radiation), consistent with combustion temperatures around 1600 °C [24,25]. These findings highlight the potential of PS as a versatile and tunable platform for advanced nanoenergetic applications, including microsafety devices, microthrusters, and precision ignition systems [19,36,37].

## 2. Materials and Methods

The fabrication of nanoporous silicon (nPS) films was performed by electrochemical anodization. P-type silicon wafers with two distinct resistivities—20 Ω·cm and 0.005 Ω·cm—were employed as substrates. To ensure reliable ohmic contact, a 350 nm aluminum layer was deposited on the backside of each wafer by thermal evaporation using a Balzers BAE 370 system operating at a base pressure of 4 × 10^−6^ Torr.

**Figure 1:**
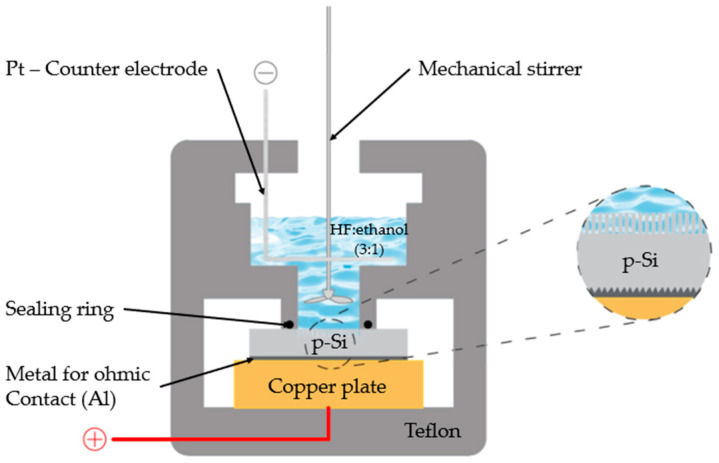
The electrochemical anodization cell.

The anodization was conducted in a Teflon electrolytic cell containing a hydrofluoric acid (HF)/ethanol solution with a volumetric ratio of 3:1, prepared from 48% aqueous HF and absolute ethanol (schematically illustrated in Figure 1). The current density was systematically varied between 50 and 200 mA cm^−2^, while the anodization time was adjusted from 10 to 25 min to tune the porosity and thickness of the resulting nPS layers.

Process parameters were systematically varied to investigate the relationship between the structural characteristics of the porous silicon (PS) films and the energetic performance of the resulting nanoenergetic composites. Current densities of 50, 75, 100, 150, and 200 mA·cm^−2^ and anodization durations of 10, 20, and 25 min (as summarized in Table 1) were employed. These conditions were selected to enable a comprehensive analysis of how anodization parameters influence pore morphology, porosity, and, consequently, the explosive behavior of PS-based devices. The overall fabrication workflow is illustrated in Figure 1. A KEITHLEY 236 programmable power supply was used to precisely control the anodization current during film formation. It is important to note that all devices were fabricated in triplicate under identical manufacturing conditions to verify the reproducibility of the fabrication process.

**Figure 2:**
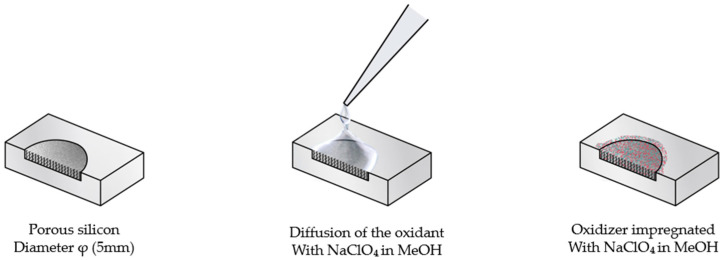
Impregnation process with sodium perchlorate (NaClO_4_) in methanol (MeOH).

The oxidant impregnation step was carried out using a 3.2 M sodium perchlorate (NaClO_4_) solution in methanol (MeOH), freshly prepared and maintained at room temperature. For each porous silicon (PS) sample, two drops of the oxidizing solution were gently dispensed onto the film surface, allowing capillary-driven diffusion of the oxidant throughout the porous network. The infiltration was performed under ambient conditions for 15 min, ensuring homogeneous NaClO_4_ distribution within the PS matrix prior to subsequent drying (Figure 2).

After impregnation, the samples were subjected to a vacuum drying process to eliminate residual solvent and stabilize the energetic composite. Drying was performed under a pressure of 27.5 mbar at a controlled temperature of 60 °C for 10 min, ensuring complete methanol evaporation and uniform distribution of sodium perchlorate within the porous matrix.

**Figure 3:**
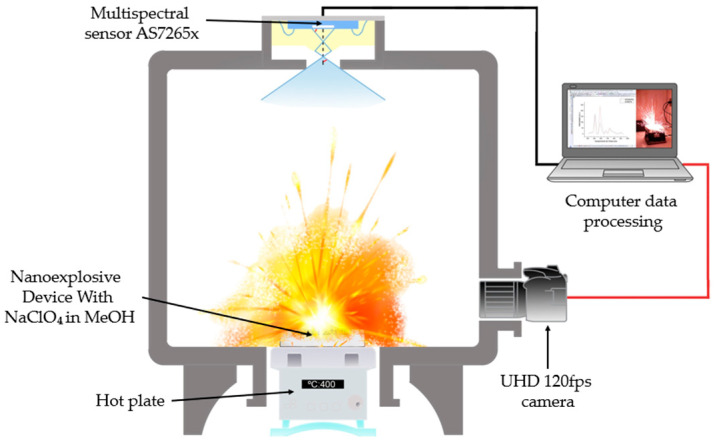
Explosion chamber with AS7265X Multispectral Sensor and 120 fps UHS camera.

The detonation experiments were performed in a hermetically sealed reaction chamber equipped with two optical access windows. The optical characterization system was mounted on one of these windows and consisted of an 18-channel multispectral sensor coupled to the chamber through an optical-fiber feedthrough. This fiber-coupled configuration enabled in situ spectral measurements while effectively isolating and protecting the multispectral sensor from the hazardous internal environment. The second access windows was used for simultaneous optical and acoustic monitoring, employing a high-resolution UHD camera (120 fps, Samsung Galaxy S24 Ultra “Samsung Electronics Co., Ltd., Suwon, Republic of Korea”). Ignition was initiated using a temperature-controlled heating plate positioned at the base of the chamber, while a thermocouple placed near the sample surface continuously recorded the ignition temperature. The overall configuration of the experimental setup is illustrated in Figure 3.

The photoluminescent emission spectrum resulting from the explosive process was acquired with the AS7265X multispectral sensor (ams OSRAM Group, Premstätten, Austria), selected for its ability to simultaneously measure 18 spectral channels (410–940 nm) with spectral resolution (±5 nm) [38]. This sensor integrates three independent subsensors (AS72651, AS72652, AS72653), each optimized for a specific spectral region (UV-Vis, Vis-NIR and NIR) [39], which operated in tandem to cover the entire range of interest. The final configuration adopted was integration time of 100.8 ms with a gain of 64×, this configuration balanced the sensitivity required for nanoburst events (μs to ms) with the thermal stability of the system, avoiding saturation of the photodiode [40].

## 3. Results and Discussion

The behavior of the SP/NaClO_4_ devices was characterized through qualitative analysis of explosion images, optical emission spectra, and acoustic signal evaluation. The results obtained from each technique are presented and discussed below.

### 3.1. Spectral Analysis of the Photoluminescent Emission of Nano-Explosive Devices

**Figure 4:**
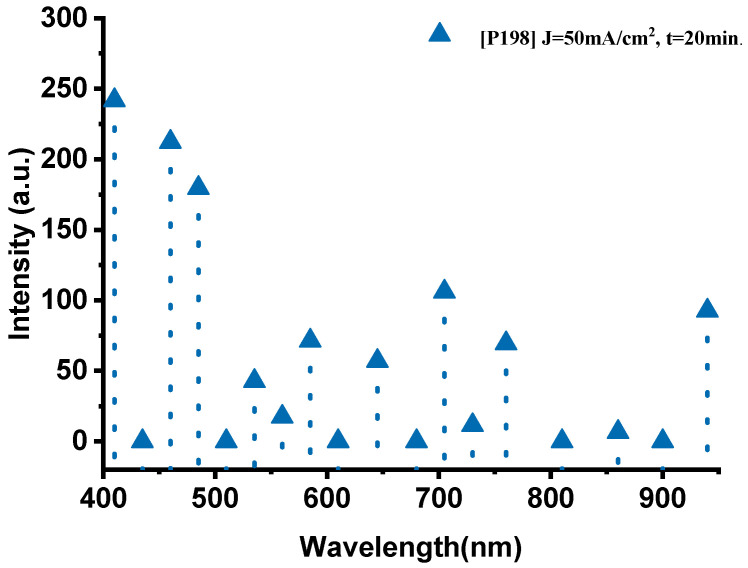
The optical emission spectrum of the P198 sample nano-explosive device.

The emission spectrum of the P198 sample, fabricated with a current density of 50 mA·cm^−2^ and an anodization time of 20 min, exhibits distinct and well-defined spectral features (Figure 4). In the visible range (400–700 nm), several prominent emission peaks were observed. The peaks at 410 nm (241.8 a.u.), 460 nm (212.3 a.u.), and 485 nm (179.5 a.u.) correspond to oxygen transitions (O-II), confirming the active participation of oxygen during combustion. In the 535–585 nm region, the spectrum displays the characteristic sodium emission lines, with a dominant peak at 535 nm (42.7 a.u.) attributed to Na-II, and additional peaks at 560 nm (17.3 a.u.) and 585 nm (71.3 a.u.) associated with Na-I transitions.

In the near-infrared (700–940 nm) region, key thermal signatures were detected. The bands at 705 nm (106 a.u.) and 760 nm (69.4 a.u.) are attributed to oxygen (O-I) transitions, whereas the strong emission at 940 nm (92.6 a.u.) corresponds to thermal radiation from the high-temperature reaction zone, indicating combustion temperatures approaching 1600 °C. The intensity of this thermal band suggests efficient energy conversion and rapid heat transfer within the PS/NaClO_4_ composite.

**Figure 5:**
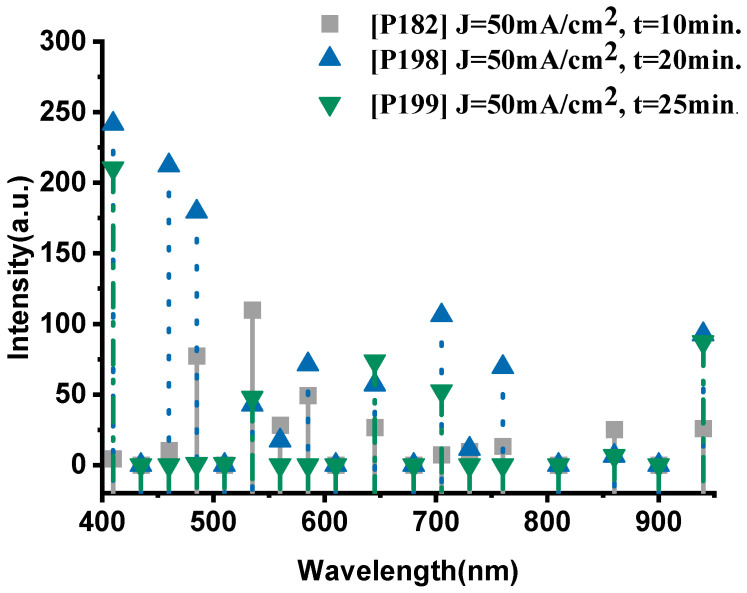
The optical emission spectra of sample P182, P198 and P199, respectively.

As shown in Figure 5, the P182 sample (anodized for 10 min) exhibits significantly weaker emissions, with characteristic peaks at 535 nm (109.9 a.u., Na-II) and 485 nm (77.4 a.u., O-II), while the thermal signal at 940 nm remains low (26 a.u.), indicating incomplete oxidation and limited energy release. Increasing the anodization time to 20 min (P198) led to a pronounced enhancement across the entire spectrum, with notably higher oxygen activity (410 nm, 241.8 a.u.; 460 nm, 212.3 a.u.) and intensified sodium lines (585 nm, 71.3 a.u.). The near-infrared emission (940 nm, 92.6 a.u.) also increased substantially, confirming more efficient combustion.

However, further extending the anodization duration to 25 min (P199) resulted in a decline in both oxygen and sodium emissions. The O-II peak at 410 nm decreased to 210.4 a.u., and the Na-II peak at 535 nm dropped to 47.8 a.u., while the thermal signal at 940 nm fell to 87.2 a.u. This trend indicates that excessive anodization time degrades the porous structure, reducing the reactive surface area and limiting oxidant–fuel interaction.

**Figure 6:**
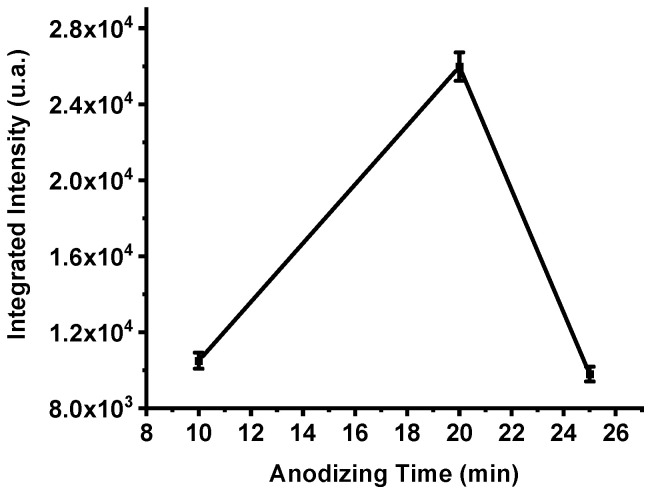
The integrated intensities of the spectra of samples P182, P198 and P199, respectively. The error bar represents the deviation in the measurement of three samples.

The integrated emission intensities of the three samples (Figure 6) confirm this behavior. The total emission of the P182 device (10 min anodization) reached 10,504 a.u., increased markedly to 25,978 a.u. for P198 (20 min), and then decreased to 9800 a.u. for P199 (25 min). These results clearly demonstrate that anodization time critically influences the porosity and energetic efficiency of PS-based nanoexplosive films. Insufficient anodization produces shallow pores and incomplete oxidant impregnation, while over-anodization enlarges pore sizes excessively, reducing the specific surface area available for oxidation. Anodization duration of approximately 20 min provides an optimal balance between oxidant diffusion depth and reactive surface density, yielding maximum energy release during combustion.

The emission spectra and the integrated-intensity trends shown in Figure 5 and Figure 6 indicate that greater film thickness (and therefore larger oxidant capacity) does not necessarily translate into higher energy release. Longer anodization times are known to increase pore size and overall porosity [41]. This coarsening tends to reduce the specific surface area available for reaction. Because combustion proceeds at exposed silicon–oxidant interfaces, a reduced reactive surface area limits the rate and completeness of the redox reaction, thereby lowering the observed optical emission and overall energy output. This mechanism explains the decline in integrated intensity observed for samples anodized beyond ~20 min.

**Figure 7:**
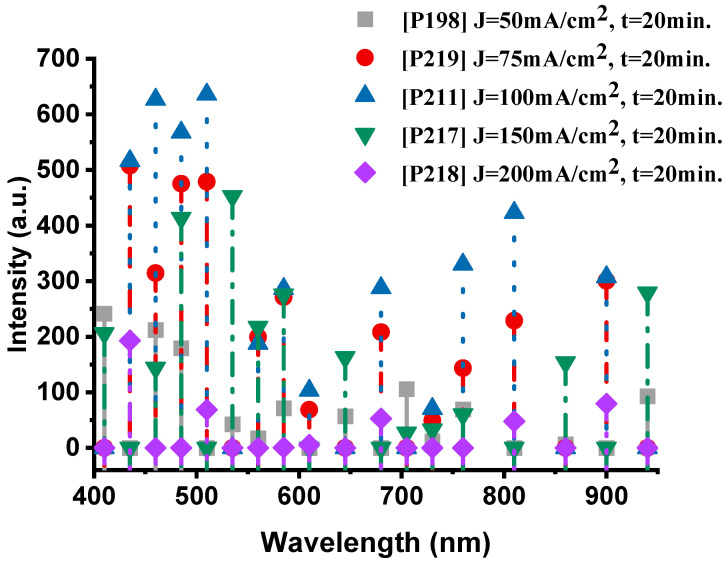
The optical emission spectra of the nanoexplosive device of samples P198, P219, P211, P217 and P218, respectively.

Figure 7 presents the emission spectra of the samples fabricated at different anodization current densities while maintaining a constant anodization time of 20 min. The P198 sample (50 mA·cm^−2^, 20 min) exhibits a well-balanced spectral profile, with pronounced oxygen (O-II) emissions at 410 nm (241.8 a.u.) and 460 nm (212.3 a.u.), accompanied by distinct sodium (Na-I) lines at 585 nm (71.3 a.u.). The thermal emission in the near-infrared region (940 nm, 92.6 a.u.) confirms efficient combustion, indicating active participation of both oxygen and sodium species in the exothermic reaction.

When the current density increases to 75 mA·cm^−2^ (P219), the oxygen emission becomes markedly stronger, particularly at 485 nm (507.6 a.u.), while sodium lines (Na-I at 585 nm, 271.1 a.u.) remain intense. This enhancement suggests that a moderate increase in current density promotes the formation of a more reactive porous structure, improving oxidant accessibility and oxygen release from NaClO_4_, while maintaining sodium’s key role in the redox process.

The P211 sample (100 mA·cm^−2^, 20 min) represents the optimal condition, exhibiting the highest overall spectral intensity. The dominant oxygen peaks at 510 nm (635.9 a.u.) and 460 nm (626.5 a.u.), together with the sodium line at 585 nm (286 a.u.), indicate vigorous combustion. The strong thermal emission centered at 810 nm (423.1 a.u.) further confirms efficient energy release and robust coupling between the oxygen and sodium reactions.

At higher current densities, this balance begins to deteriorate. In the P217 sample (150 mA·cm^−2^), oxygen emissions remain relatively strong (535 nm, 452.7 a.u.), but sodium peaks (585 nm, 275.8 a.u.) show a slight decline. The thermal signal (940 nm, 279.1 a.u.) is still present but noticeably weaker than in P211, suggesting that excessive current densities begin to degrade the PS structure, thereby reducing sodium’s contribution to the combustion process.

Finally, the P218 sample (200 mA·cm^−2^) exhibited the lowest performance, characterized by weak emissions from both species—oxygen at 435 nm (192.9 a.u.) and the absence of significant sodium (Na-I) peaks. The corresponding thermal emission at 900 nm (79.4 a.u.) was also the weakest among all samples, indicating that excessive current density compromises combustion efficiency. This degradation is likely caused by structural damage to the porous network, which restricts oxidant infiltration and reduces the number of reactive surface sites.

In the range of 50–100 mA·cm^−2^, oxygen (O-II) and sodium (Na-I) emissions act synergistically, reaching their maximum intensities for the P211 sample (100 mA·cm^−2^). At higher current densities (>100 mA·cm^−2^), sodium emission intensity decreases progressively, while oxygen transitions also lose efficiency, resulting in less effective combustion. Therefore, a current density of approximately 100 mA·cm^−2^ can be identified as the optimum condition for maximizing the participation of both elements in the nanoenergetic reaction.

**Figure 8:**
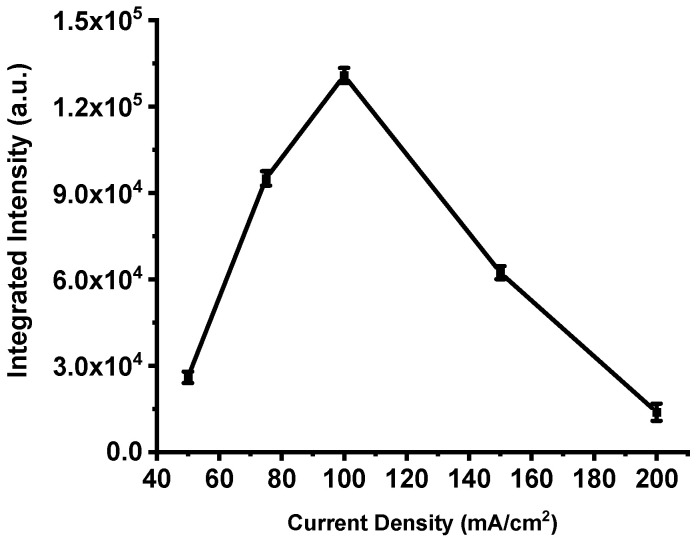
Integrated intensities of the spectra shown in Figure 7. The error bar represents the deviation in the measurement of three samples.

This trend is clearly illustrated in Figure 8, which presents the integrated emission intensity for each device. The total intensity increases steadily from the film produced at 50 mA·cm^−2^, peaks at 100 mA·cm^−2^, and then declines sharply for higher current densities. These results confirm that excessive anodization current deteriorates the structural and energetic balance of the porous silicon matrix, leading to a measurable loss in combustion performance.

The foregoing results demonstrate that anodization current density strongly controls pore morphology in PS films: both pore size and overall porosity tend to increase with increasing current density. This trend has been documented in several studies [27,42]. Because the energetic performance of PS-based nanoexplosive devices is governed by the available reactive surface, and because specific surface area generally decreases as porosity coarsens [43], higher current densities can ultimately reduce the effective reaction area and thereby impair device performance.

Figure 7 and Figure 8 show that the energy output in this work rises with current density up to a well-defined maximum and then falls off sharply. If performance depended solely on specific surface area, one would expect a monotonic relationship; the observed non-monotonic behavior therefore indicates additional competing effects [25,37,44,45,46]. A key secondary factor is the oxidant fill factor—the fraction of pore volume actually occupied by the impregnated oxidizer. Very fine pores (high specific surface, e.g., ≈600 m^2^/cm^3^ with ~2 nm pores) typically exhibit low fill factors (~20%), whereas coarser pores (e.g., ~8 nm pores and ≈200 m^2^/cm^3^) can attain fill factors approaching ~80%. Consequently, there exists an optimum pore morphology that balances a high density of reactive Si sites (proportional to specific surface area) against sufficient pore volume to host the oxidant.

Previous reports identify this optimum near pore diameters of ~4–4.8 nm and specific surface areas on the order of ≈300 m^2^/cm^3^ [47]. This framework explains the data presented here: the integrated emission increases from ~25,698 a.u. for P198 (50 mA·cm^−2^) to a maximum of ~130,786 a.u. for P211 (100 mA·cm^−2^), consistent with an optimal oxidant filling factor at intermediate porosity. At higher current densities, although the fill factor may continue to increase, the concomitant loss of specific surface area (and, in extreme cases, collapse of the porous network) reduces the number of accessible reactive sites and degrades combustion efficiency.

### 3.2. Analysis of Acoustic Noise Signals Originated by the Explosion Process of Nanoexplosive Devices

**Figure 9:**
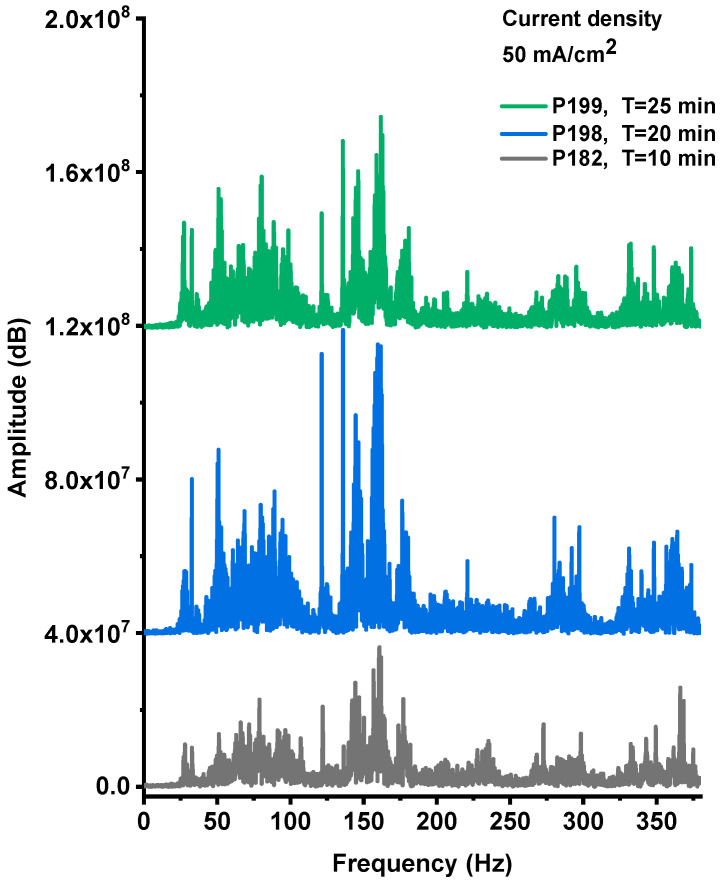
The Fourier spectra of the acoustic noise signal from P182, P198, P199 devices, respectively.

**Figure A1:**
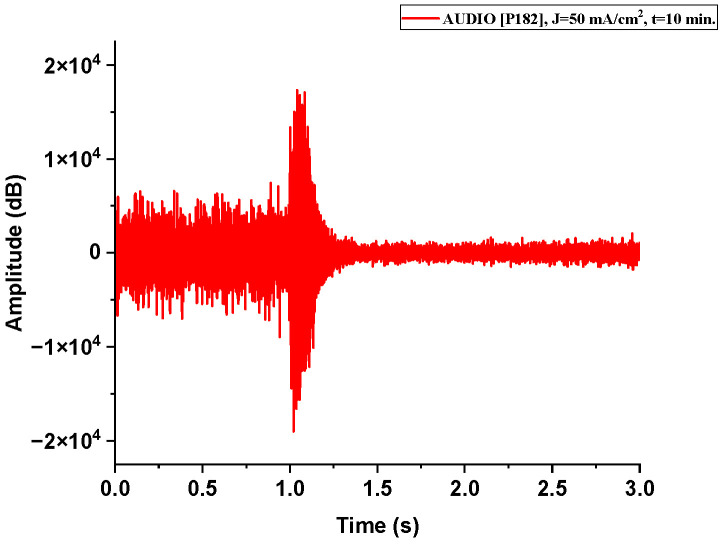
The acoustic signal originated from Sample P182 explosion.

**Figure A2:**
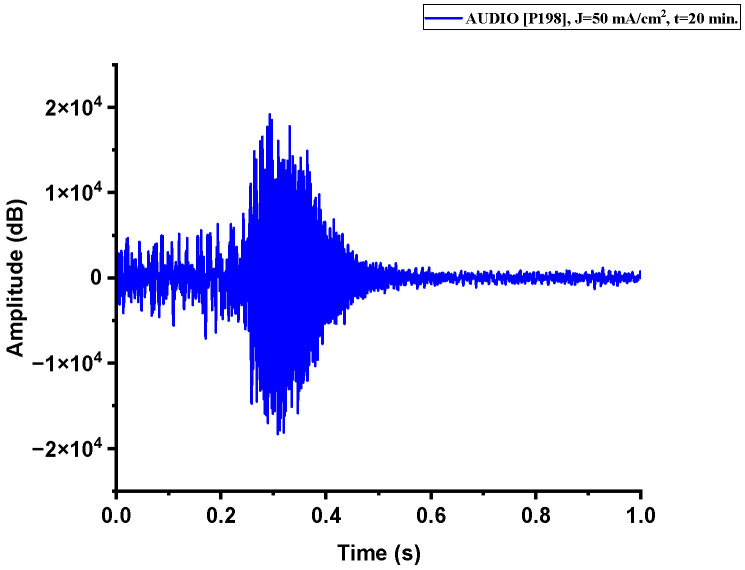
The acoustic signal originated from Sample P198 explosion.

**Figure A3:**
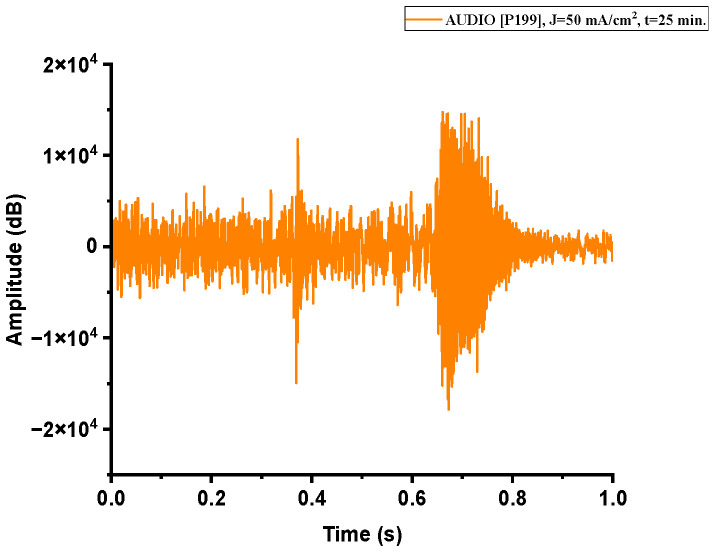
The acoustic signal originated from Sample P199 explosion.

The explosive events of the PS/NaClO_4_ devices produced both optical emission and acoustic shock waves. Figure A1, Figure A2 and Figure A3 (Appendix A) present the acoustic noise signals recorded from explosions of devices fabricated with PS films anodized at a current density of 50 mA·cm^−2^ for 10, 20, and 25 min, respectively. The recorded signals exhibit a characteristic wave-packet profile, defined by an envelope with a distinct maximum amplitude and a finite duration. The Fourier spectra corresponding to these signals are shown in Figure 9.

The spectral analysis revealed that the explosive events produced acoustic emissions within the 20–380 Hz frequency range, which is characteristic of detonation and rapid combustion phenomena. The acoustic spectra shown in Figure 9 display multiple spectral lines whose amplitudes vary consistently with both the intensity and duration of the time-domain noise bursts, as well as with the photoluminescent emission observed during the explosions. The highest-amplitude spectral components were recorded for the sample anodized for 20 min, indicating that this processing condition yields the most energetic and efficient combustion behavior.

**Figure 10:**
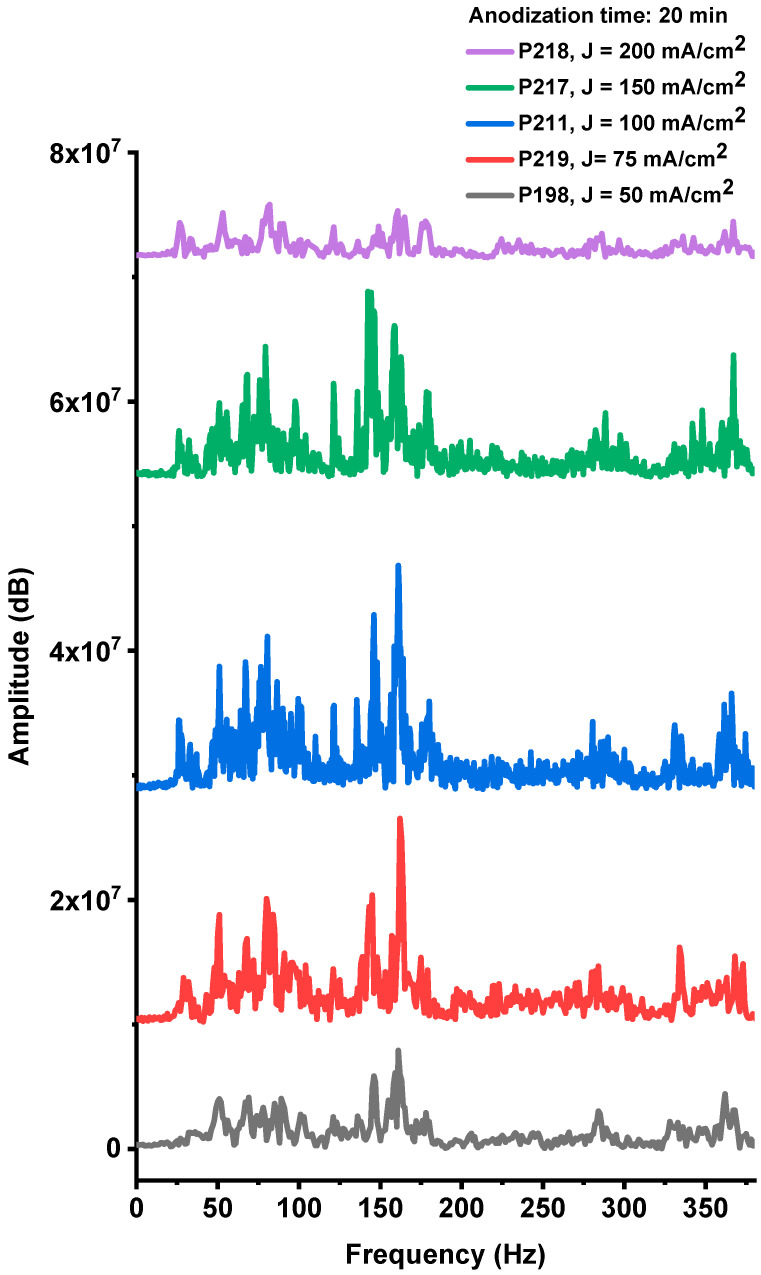
Fourier spectra of the acoustic noise signals corresponding to samples obtained with the same anodizing time (t = 20 min) and different current densities 50, 75, 100, 150 and 200 mA/cm^2^ (samples P198, 219, 211, 217, 218, respectively).

**Figure A4:**
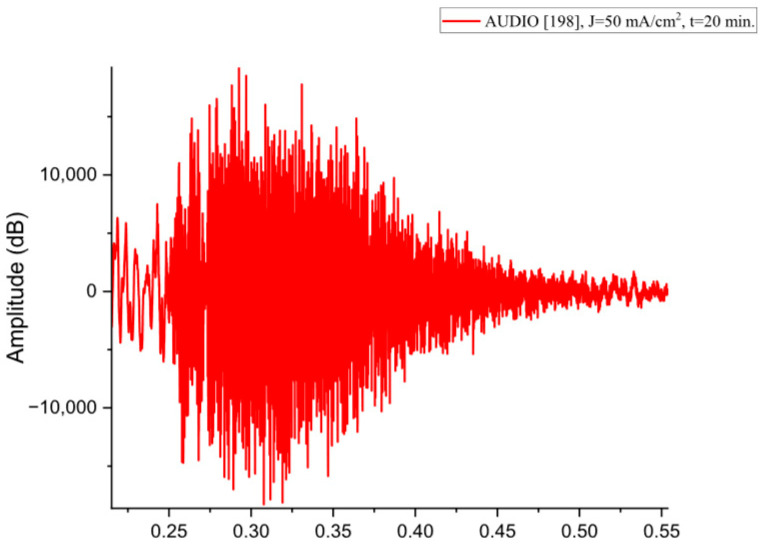
The acoustic signal originated from Sample P198 explosion.

**Figure A5:**
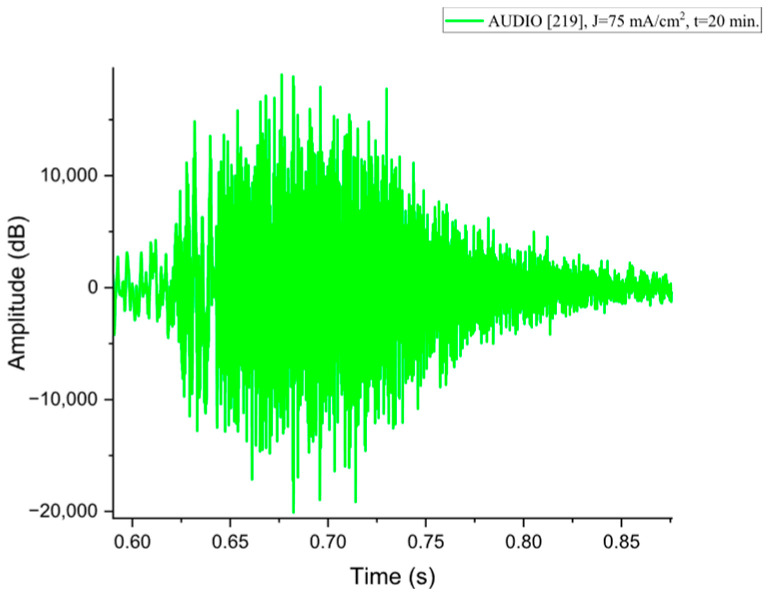
The acoustic signal originated from Sample P219 explosion.

**Figure A6:**
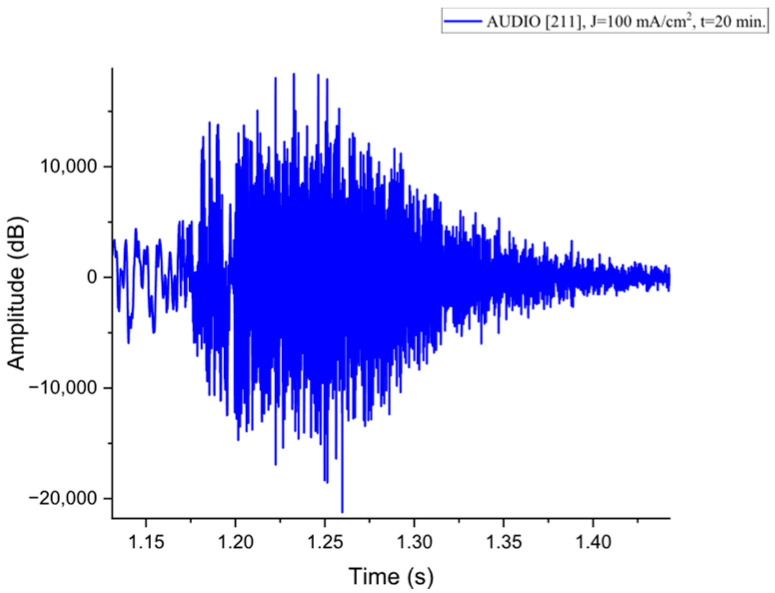
The acoustic signal originated from Sample P211 explosion.

**Figure A7:**
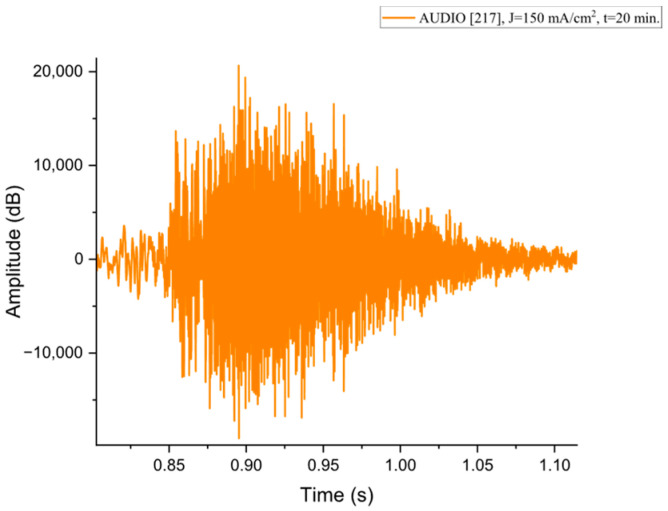
The acoustic signal originated from Sample P217 explosion.

**Figure A8:**
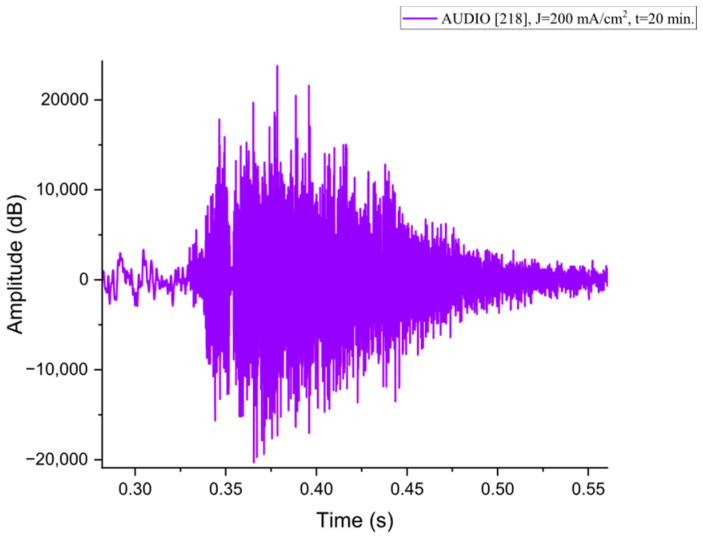
The acoustic signal originated from Sample P218 explosion.

The acoustic responses generated during the explosions of devices fabricated on PS substrates with different anodization current densities (P198, P219, P211, P217, and P218), while maintaining a constant anodization time of 20 min, are presented in Figure A4, Figure A5, Figure A6, Figure A7 and Figure A8 (Appendix A). The corresponding frequency-domain representations, obtained from Fourier spectral analysis (Figure 10), reveal prominent low-frequency components. As previously discussed, these components originate from the shock waves produced during the explosive combustion of the PS/NaClO_4_ composites.

The acoustic spectra of these samples exhibit multiple spectral lines within the 20–380 Hz band (Figure 10). Signal amplitudes vary systematically with the anodization current density used during fabrication, with the P211 sample (100 mA·cm^−2^) showing the largest spectral amplitudes. This acoustic response correlates closely with the optical-emission results, indicating that both diagnostics capture the same underlying combustion strength. Together, the acoustic and optical data identify an optimal anodization current density near 100 mA·cm^−2^, which maximizes device performance. Deviations from this condition—either lower or higher current densities—reduce explosive output, reflecting the trade-off between oxidant fill factor and specific surface area described above.

The surface chemistry of porous silicon (PS) critically influences the ignition behavior of PS/NaClO_4_ composites. After formation and drying, PS surfaces are terminated by a mixture of species—Si-H_n_, Si-OH, Si-O-Si, and Si-OR (R = organic/siloxane residues)—which leave a population of undercoordinated (dangling) Si sites exposed [48,49]. These dangling bonds act as preferential ignition loci, while the coexisting oxygenated and siloxane terminations form a partially passivating layer that suppresses spontaneous redox reactions with the impregnated perchlorate [29,49]. As a result, the composite remains metastable at ambient conditions; by contrast, a surface dominated by reactive hydride terminations (Si-H_n_) could facilitate local acidification and formation of stronger oxidants (e.g., HClO_4_), increasing the likelihood of uncontrolled reaction. The mixed termination therefore establishes a practical balance between storage stability and on-demand reactivity, enabling controlled ignition via localized thermal heating (as used here) or targeted optical excitation [14].

## 4. Conclusions

In this work, nanoporous silicon (PS) films fabricated by electrochemical anodization were developed into reproducible nanoenergetic devices. The PS layers functioned both as the solid fuel and as a host matrix for the oxidizer (NaClO_4_), enabling rapid, and spatially confined energy release. Devices prepared on p-type silicon wafers (resistivity 10–20 Ω·cm) exhibited superior energetic performance compared with those fabricated on heavily doped p^+^/n^+^ substrates, a difference attributed to the larger accessible reactive surface area in lightly doped material.

Impregnation with a 3.2 M NaClO_4_ solution in methanol produced homogeneous composites with high oxidizer loading and consistent performance after vacuum drying. Comprehensive characterization by optical emission spectroscopy and acoustic analysis showed that visible emissions (400–750 nm) correspond to O and Na electronic transitions, while near-infrared emission (800–950 nm) is dominated by thermal radiation consistent with combustion temperatures approaching ~1600 °C. Acoustic bursts lasted ~140–335 ms** and contained dominant spectral components in the 20–380 Hz range.

Energetic output correlated strongly with PS morphology: films anodized for ≈20 min provided the best compromise between oxidant uptake and reactive surface area, while shorter anodization time produced pores too small for efficient oxidant impregnation and longer anodization time coarsened the structure, reducing specific surface area. Likewise, a current density of ~100 mA·cm^−2^ yielded optimal performance, balancing porosity and fill factor to maximize combustion efficiency.

These findings define practical processing–structure–property relationships for PS/NaClO_4_ nanoenergetics and provide optimized fabrication parameters for applications that require controlled micro-scale energy release, including microsensors, precise ignition elements, and micropropulsion devices.

## Figures and Tables

**Table 1:** Anodization parameters for the synthesis of nanoporous silicon (nPS) in HF/ethanol solution.

Sample	Type of the Si	Current Density J (mA/cm^2^)	Anodization Time t (min.)	Electrolyte Solution HF:Ethanol
P182	P	50	10	3:1
P198	P	50	20	3:1
P199	P	50	25	3:1
P219	p	75	20	3:1
P211	P	100	20	3:1
P217	P	150	20	3:1
P218	P	200	20	3:1

## Data Availability

The data supporting the findings of this study are available within the article.

## Appendix A

The acoustical sound signal release from the explosion process of the devices fabricated with PS film that was obtained with the same current density (50 mA/cm^2^) at different anodization times: 10, 20 and 25 min respectively.

The acoustical sound signal release from the explosion process of the devices fabricated with PS film that was obtained with the same current density (50 mA/cm^2^) at different anodization times: 10, 20 and 25 min respectively.
